# Mouse d-Amino-Acid Oxidase: Distribution and Physiological Substrates

**DOI:** 10.3389/fmolb.2017.00082

**Published:** 2017-12-04

**Authors:** Reiko Koga, Yurika Miyoshi, Hiroaki Sakaue, Kenji Hamase, Ryuichi Konno

**Affiliations:** ^1^Faculty of Pharmaceutical Sciences, Fukuoka University, Fukuoka, Japan; ^2^Graduate School of Pharmaceutical Sciences, Kyushu University, Fukuoka, Japan; ^3^Department of Biochemistry, Tokyo University of Pharmacy and Life Sciences, Hachioji, Japan; ^4^Department of Pharmacological Sciences, International University of Health and Welfare, Ohtawara, Japan

**Keywords:** d-amino-acid oxidase, mouse, distribution, physiological substrates, d-serine

## Abstract

d-Amino-acid oxidase (DAO) catalyzes the oxidative deamination of d-amino acids. DAO is present in a wide variety of organisms and has important roles. Here, we review the distribution and physiological substrates of mouse DAO. Mouse DAO is present in the kidney, brain, and spinal cord, like DAOs in other mammals. However, in contrast to other animals, it is not present in the mouse liver. Recently, DAO has been detected in the neutrophils, retina, and small intestine in mice. To determine the physiological substrates of mouse DAO, mutant mice lacking DAO activity are helpful. As DAO has wide substrate specificity and degrades various d-amino acids, many d-amino acids accumulate in the tissues and body fluids of the mutant mice. These amino acids are d-methionine, d-alanine, d-serine, d-leucine, d-proline, d-phenylalanine, d-tyrosine, and d-citrulline. Even in wild-type mice, administration of DAO inhibitors elevates D-serine levels in the plasma and brain. Among the above d-amino acids, the main physiological substrates of mouse DAO are d-alanine and d-serine. These two d-amino acids are most abundant in the tissues and body fluids of mice. d-Alanine derives from bacteria and produces bactericidal reactive oxygen species by the action of DAO. d-Serine is synthesized by serine racemase and is present especially in the central nervous system, where it serves as a neuromodulator. DAO is responsible for the metabolism of d-serine. Since DAO has been implicated in the etiology of neuropsychiatric diseases, mouse DAO has been used as a representative model. Recent reports, however, suggest that mouse DAO is different from human DAO with respect to important properties.

## Introduction

d-Amino-acid oxidase (DAO, DAAO, EC 1.4.3.3) catalyzes the oxidative deamination of d-amino acids, generating the corresponding 2-oxo acids (α-keto acids) along with hydrogen peroxide and ammonia. It displays a wide spectrum of substrate specificity, degrading many neutral and basic d-amino acids. DAO exists in a wide range of organisms, such as fungi, mollusks, insects, fish, amphibians, reptiles, birds, and mammals (Meister, 1965; Pollegioni et al., 2007). It has important roles in catabolism, elimination, detoxification, and re-utilization of d-amino acids, as well as in biophylaxis and neuromodulation depending on the organisms (Pollegioni et al., 2007; Ohide et al., 2011). In mammals, DAO is mainly present in the kidney, liver, and brain.

Mice have been used in a variety of studies involving d-amino acids, such as the elucidation of the physiological functions of DAO, D-aspartate oxidase, and serine racemase, the nutritional utilization of d-amino acids, the elucidation of etiology of neuropsychiatric diseases involving D-serine, the development of DAO inhibitors, and so on [Konno et al. (2008); Brückner and Fujii (2011); Ferraris and Tsukamoto (2011), and Yoshimura et al. (2016)]. Interestingly, mouse DAO has some unique features. It is a 39 KDa protein consisting of 345 amino acid residues (Tada et al., 1990). The number of the constituent amino acids is two less than that of human DAO due to the deletion of amino acid residues during the evolution of rodents (Ohide et al., 2014). The 27th and 183rd residues present in human DAO is missing in mouse DAO (Momoi et al., 1988; Tada et al., 1990; Tsuchiya et al., 2003). However, 81% of the amino acid residues in the sequence are identical between the two enzymes. The 3D structure of human DAO has been determined but not of mouse DAO (Kawazoe et al., 2006; Pollegioni et al., 2007). Therefore, it is not clear how these deletions cause conformational changes affecting enzymatic property and susceptibility to DAO inhibitors to compare with human DAO. In addition to the amino acid deletions, DAO is not present in the mouse liver, in contrast to other animals (Meister, 1965; Konno et al., 1997).

D-Serine is one of the good substrates of DAO and is abundantly present in the mammalian brains. It functions as a co-agonist of NMDA-subtype of glutamate receptors and potentiates NMDA receptor activity. NMDA receptors are involved in the higher brain function. DAO modulates NMDA receptor activity via the degradation of D-serine. Therefore, aberrant DAO activity is implicated in the etiology of neuropsychiatric diseases, such as schizophrenia (Chumakov et al., 2002) and amyotrophic lateral sclerosis (Mitchell et al., 2010). Mutant mice lacking DAO have been used as an animal model for the elucidation of the etiology of these diseases (Sakaue et al., 2016).

DAO is attracting the attention of researchers owing to its implication in the neuropsychiatric diseases. In this review, we focus on recent findings relating to the distribution and physiological substrates of mouse DAO. For human DAO, see the reviews, “Biochemical properties of human DAAO” by Murtas et al. and “Structural features of human DAAO” by Molla in this topic.

## Distribution of mouse DAO (Table 1)

DAO has been detected in peroxisomes of cells of the proximal tubules (Usuda et al., 1986), in peroxisomes of hepatocytes (Perotti et al., 1987), and in the microperoxisomes of astrocytes in the cerebellum (Arnold et al., 1979). These findings were obtained in rats, but the presence of DAO has also been reported in various mouse organs and tissues.

**Table 1:** Distribution and supposed functions of mouse DAO.

**Organ/Tissue/Cells**	**Supposed function**
Kidney (proximal tubules)	Elimination
	Re-utilization
Brain (cerebellum, brainstem)	Modulation of neurotransmission
	Cerebellar formation?
Spinal cord	Modulation of neurotransmission?
Neutrophils	Biophylaxis
Retina	Modulation of neurotransmission?
	Retina formation?
Small intestine	Biophylaxis

### Kidney

The most DAO-rich organ is the kidney in mice, as well as in other animals. DAO activity was detected in the mouse kidney fairly earlier (Meister, 1965), and the presence of DAO in the peroxisomes of mouse renal cells was reported subsequently (Verity et al., 1967; Goeckermann and Vigil, 1975; Konno et al., 1991). Konno and Yasumura (1983), Wang and Zhu (2003), and Zhao et al. (2008) measured DAO activity in the mouse kidney. The enzyme activity was higher in the male mice than in females (Konno and Yasumura, 1983). DAO mRNA was detected in the kidney and brain (Tada et al., 1990) and in the epithelial cells of the proximal tubules (Koibuchi et al., 1995), and immunoblotting experiments showed the presence of DAO protein in the kidney (Shibuya et al., 2013). Enzyme histochemistry also showed DAO activity in proximal tubule cells (Sasabe et al., 2014b). Therefore, administration of the suicide substrate (D-propargylglycine) of DAO caused damage in the straight part of the proximal tubule (Konno et al., 2000). These experimental data are consistent with those obtained in other animal species.

### Liver

Although the liver is the second most DAO-rich organ in other animals, it was believed that the mouse liver did not have DAO (Meister, 1965). However, some reports appeared to indicate the presence of DAO in the mouse liver (Nagata and Akino, 1988; Nagata et al., 1988; D'Aniello et al., 1993). Contrary to these reports, Tada et al. (1990) could not detect DAO mRNA in the liver by northern hybridization. Konno et al. (1997) could not prove DAO enzyme activity, the presence of DAO protein by immunoblotting, or DAO mRNA by northern hybridization and reverse-transcription PCR in the livers of BALB/c or ddY/DAO^+^ mice. Wang and Zhu (2003) similarly could not detect DAO activity or DAO transcript in the livers of C57BL/6N mice. Xin et al. (2010) were unable to detect DAO activity in the liver homogenate of Swiss mice or ddY/DAO^+^ mice, and Shibuya et al. (2013) did not detect DAO protein by immunoblotting in the livers of C57BL/6N mice. Therefore, it seems beyond doubt that DAO is not present in the mouse liver. Why the mouse exceptionally lacks DAO in the liver is an interesting question.

### Brain

The presence and distribution of DAO in brain regions has been mainly determined in rats. In mice, Goldstein (1966) detected strong DAO activity in the cerebellum, slight activity in the medulla, and trace activity in the midbrain but not in the forebrain. DAO activity was also detected in the whole brains of mice (Konno and Yasumura, 1984a), where the activity was approximately 1/20 of that of the kidney. Tada et al. (1990) detected DAO mRNA in the brain by northern hybridization. Wang and Zhu (2003) detected DAO activity in the cerebellum but not in the cortex or striatum. Hamase et al. (2006a) detected DAO activity in the cerebellum, medulla oblongata, and midbrain, but not in the cerebrum, hippocampus, hypothalamus, pituitary gland, or pineal gland.

Using enzyme histochemistry, Sasabe et al. (2012) detected strong enzymatic activity in the cerebellum, and weak activity in the pons and medulla oblongata, but not in the forebrain. They also found the presence of DAO activity in the astrocytes of the reticulospinal tract. Shibuya et al. (2013) found DAO protein in the cerebellum by immunoblotting.

Schweimer et al. (2014) found prominent immunoreactivity against DAO in the Bergman glia in the molecular layer of the cerebellum. They also found immunoreactivity in astrocytes of ventral tegmental area, where dopamine neurons exist densely. Surprisingly, they also observed some immunoreactivity in neurons.

Using an enzyme activity assay, Sasabe et al. (2014a) confirmed DAO distribution observed in 2012. Further, they examined DAO distribution in detail using enzyme histochemistry. Strong activity was detected in the molecular layer, mild activity in white matter, and patchy activity in the granular layer of the cerebellum. Low DAO activity was seen in the midbrain (ventral tegmental area), pons (reticular formation and medial lemniscus), and medulla oblongata (reticular formation). Among the neuronal pathways, strong DAO activity was detected in reticulospinal tracts and ponto/olivocerebellar fibers. Surprisingly, they found that human DAO was distributed more widely in the central nervous system, including the cerebrum and hippocampus, compared with mouse DAO.

### Spinal cord

Goldstein (1966) detected DAO activity in the spinal cords of mice. In recent years, Lu et al. (2012) also found the presence of DAO in the spinal cord, the activity of which was completely inhibited by subcutaneous injection of a DAO inhibitor. Sasabe et al. (2012) detected DAO activity and DAO protein by enzymatic assay and immunoblotting, respectively, in the homogenate of spinal cord. Using enzyme histochemistry, they observed DAO reaction in the lamina VIII and IX of ventral spinal gray matter in the anterior column. Ma et al. (2015) observed that subcutaneous injection of morphine induced spinal astroglial activation with increased gene expression of DAO.

### Neutrophils

As in human and porcine neutrophils, DAO was found to be present in mouse neutrophils (Nakamura et al., 2012). DAO, together with myeloperoxidase, produced hypochlorous acid and had an important role in the antimicrobial system. Therefore, mice lacking DAO activity were more susceptible to *Staphylococcus aureus* infection than wild-type mice. Tuinema et al. (2014) observed that the ability of neutrophils to kill *Salmonella* was impaired by a DAO inhibitor, confirming the involvement of DAO in their bactericidal activity.

### Retina

Gustafson et al. (2013) found the presence of DAO in the inner plexiform layer of the mouse retina. This DAO reduces the D-serine level in the retina and affects the ratio of *N*-methyl-D-aspartate (NMDA) and α-amino-3-hydroxy-5-methyl-4-isoxazolepropionic acid (AMPA) receptors during retinal development (Romero et al., 2014).

### Small intestine

Sasabe et al. (2016) recently found the presence of DAO in enterocytes and goblet cells of small intestine in mice by enzyme activity assays and immunoblotting. Some DAO molecules were excreted into mucus. This DAO also possesses bactericidal activity via the production of hydrogen peroxide. Accordingly, the composition of microflora in the intestinal tract in wild-type mice was shown to be different from that in DAO^G181R^ mice lacking DAO activity.

### Tissue-specific expression

As described above, mouse DAO is present in the kidney, hindbrain, spinal cord, neutrophils, and small intestine, but not in the liver or forebrain. Little is known about the regulation of the expression of the DAO gene, although presumably there are some mechanisms that strictly stimulate or suppress gene expression. Dietrich et al. (2008) and Luks et al. (2017) found that administration of propiverine (which is used to treat overactive bladder) caused massive cytosolic and nuclear accumulation of DAO in the epithelial cells of the proximal tubules of rat kidney. Jagannath et al. (2017) found that *DAO* CpG sites in the cerebellum of human brains were significantly more methylated than those in the frontal cortex. These findings may help to elucidate the mechanism of DAO gene expression in specific tissues and organs.

## Physiological substrates (Table 2)

Since DAO has wide substrate specificity, many d-amino acids are oxidatively deaminated. For the determination of physiological substrates of mouse DAO, comparisons between mutant mice lacking DAO activity and wild-type mice are very useful. The substances that accumulate in the mutant mice can be considered to be candidates for the physiological substrates. In other words, such substances are considered to be constantly degraded by DAO in the wild-type mice.

**Table 2:** Physiological substrates of mouse DAO.

**Substrate**	**Source**
D-Alanine	Bacteria
D-Serine	Synthesis by serine racemase
D-Methionine	Food
D-Leucine	Food/bacteria?
D-Proline	Food/bacteria?
D-Phenylalanine	Food/bacteria/by-product?
D-Tyrosine	Food/bacteria/by-product?
D-Citrulline	Food/bacteria/by-product?

Five mouse strains that do not have DAO activity have been established so far and have been used in various studies. The ddY/DAO^−^ mice carry a missense mutation (Gly181Arg) that causes a complete loss of DAO activity (Konno and Yasumura, 1983; Sasaki et al., 1992). Labrie et al. (2009), Sasabe et al. (2012), and Koga et al. (2016b) independently crossed mutant ddY/DAO^−^ mice with C57BL/6J mice and continuously backcrossed their offspring to C57BL/6J mice in order to transfer the Gly181Arg mutation to a C57BL/6J genetic background. Consequently, three inbred strains have been separately established: the *Dao1*^*G181R*^ strain (Labrie et al., 2009), the ^B6^DAO^−/−^ strain (Sasabe et al., 2012), and the B6DAO^−^ strain (Koga et al., 2016b). More recently, a knockout strain (DAO-KO) has been generated using genetic engineering techniques (Rais et al., 2012). The DAO-KO mice have a deletion of exon 6-9 of the *Dao1* gene in a 129SvEV genetic background.

Many researchers have shown that large amounts of d-amino acids accumulate in the organs and body fluids of the mutant mice (Hamase et al., 2005; Konno et al., 2010).

### D-methionine

The urine of ddY/DAO^−^ mice lacking DAO activity contained nearly three times more D-methionine than that of wild-type ddY/DAO^+^ mice (Konno et al., 1988). This was because the commercial mouse diet was supplemented with DL-methionine to meet the nutritional requirements of experimental animals. As the ddY/DAO^−^ mice could not metabolize the supplemental D-methionine by using DAO, they excreted it into urine. Therefore, as expected, the ddY/DAO^−^ mice fed the mouse diet supplemented with L-methionine in place of DL-methionine excreted the same level of methionine into urine as did the wild-type ddY/DAO^+^ mice (Konno et al., 1988).

### D-alanine

Urine of ddY/DAO^−^ mice contained 13 times more D-alanine than that of wild-type ddY/DAO^+^ mice (Konno et al., 1989). When ddY/DAO^−^ mice were made germ-free, they excreted very low levels of D-alanine into urine. When they were made gnotobiotic by inoculation of several species of intestinal bacteria, they again excreted a large amount of D-alanine into urine. Therefore, the D-alanine in the urine was determined to originate from intestinal bacteria (Konno et al., 1993). It is well known that bacteria have D-alanine in their cell walls as an essential component of peptidoglycan. Karakawa et al. (2013) also observed that the plasma of SPF-ICR mice possessing DAO contained more D-alanine than that of germ-free ICR mice, confirming the intestinal bacterial origin of D-alanine in the plasma.

In addition to urine, the serum, kidney, pancreas, and brain of ddY/DAO^−^ mice have been found to contain more D-alanine than those of ddY/DAO^+^ mice (Table 3; Nagata et al., 1992; Hashimoto et al., 1993; Morikawa et al., 2001, 2007; Miyoshi et al., 2009). The D-alanine levels were increased rather uniformly in all areas of the brain (cerebrum, hippocampus, olfactory bulb, hypothalamus, pituitary gland, pineal gland, cerebellum, and medulla oblongata). It seems that an elevated level of D-alanine in the blood caused the high levels of D-alanine in these brain regions. Indeed, oral administration of D-alanine to the ddY/DAO^−^ mice increased D-alanine to much higher levels in the serum, liver, kidney, cerebrum, and brain (Nagata et al., 1994; Morikawa et al., 2007).

**Table 3:** Amounts of D-alanine in the tissues and physiological fluids of wild-type mice and mutant mice lacking DAO activity.

**Tissues or physiological fluids**	**Wild-type mice (nmol/g or mL)**	**Mutant mice (nmol/g or mL)**	**References**
Cerebrum	12.4	63.7^**^	Morikawa et al., 2001
	4.6	24.6^**^	Miyoshi et al., 2009
Hippocampus	11.4	61.7^**^	Morikawa et al., 2001
	3.6	24.1^**^	Miyoshi et al., 2009
Olfactory bulb	3.6	17.5^**^	Miyoshi et al., 2009
Hypothalamus	9.0	52.6^**^	Morikawa et al., 2001
	5.7	34.9^**^	Miyoshi et al., 2009
Pituitary gland	29.1	81.2^**^	Morikawa et al., 2001
	15.1	80.1^**^	Miyoshi et al., 2009
Pineal gland	36.5^a^	60.1^a^	Morikawa et al., 2001
Cerebellum^#^	10.9	79.6^**^	Morikawa et al., 2001
	0.4	36.8^**^	Miyoshi et al., 2009
Medulla oblongata^#^	4.6	59.4^**^	Morikawa et al., 2001
	1.0	39.9^**^	Miyoshi et al., 2009
Serum	8.8	134.6^**^	Morikawa et al., 2001
	10.9	47.6^**^	Miyoshi et al., 2009
Urine	58.5	2, 079.0^**^	Miyoshi et al., 2009
Liver	17.5	71.9^**^	Miyoshi et al., 2009
Kidney^#^	5.8	935.2^**^	Miyoshi et al., 2009
Pancreas	109.4	486.6^**^	Miyoshi et al., 2009

*The literature since 2001 has been compiled in this table. The values represent means and are obtained from ddY/DAO^−^ mice*.

a *Values represent pmol/whole tissues*.

# *denotes tissues where DAO is present*.

** *P < 0.01 compared with wild-type mice*.

D-Alanine derived from bacteria serves as the substrate of DAO, which is part of the biophylaxis system as described in the neutrophils and intestine. D-Alanine may also serve as a co-agonist of NMDA receptors, and its concentration is controlled by DAO.

### D-serine

Large amounts of D-serine were detected in the forebrains of mammals (Hashimoto et al., 1993; Hashimoto and Oka, 1997). Brain D-serine is mostly synthesized from L-serine by serine racemase (Wolosker et al., 1999; Horio et al., 2011; Miyoshi et al., 2012). It acts as a co-agonist of NMDA-subtype glutamate receptors and modulates neurotransmission (Mothet et al., 2015; Wolosker et al., 2016). The ddY/DAO^−^ and ddY/DAO^+^ mice did not show any significant differences in D-serine content in the cerebrum, hippocampus, olfactory bulb, hypothalamus, or pituitary gland. However, D-serine content in the cerebellum, medulla oblongata, and spinal cord were significantly higher in ddY/DAO^−^ mice than in ddY/DAO^+^ mice (Table 4; Hashimoto et al., 1993; Morikawa et al., 2001; Miyoshi et al., 2009, 2012). The latter brain regions are where DAO exists in wild-type mice. The differences in the amounts of D-serine and other d-amino acids in the brain regions of ddY/DAO^−^ and ddY/DAO^+^ mice have been critically compared and reviewed by Verrall et al. (2010). Hamase et al. (2005) and Yamanaka et al. (2012) have also summarized the differences.

**Table 4:** Amounts of D-serine in the tissues and physiological fluids of wild-type mice and mutant mice lacking DAO activity.

**Tissues or physiological fluids**	**Wild-type mice (nmol/g or mL)**	**Mutant mice (nmol/g or mL)**	**References**
Cerebrum	423.2	424.5	Morikawa et al., 2001
	300.0	306.1	Miyoshi et al., 2009
Hippocampus	341.5	300.3	Morikawa et al., 2001
	268.8	273.3	Miyoshi et al., 2009
Olfactory bulb	104.4	98.1	Miyoshi et al., 2009
Hypothalamus	105.2	147.1	Morikawa et al., 2001
	127.4	175.5^**^	Miyoshi et al., 2009
Pituitary gland	3.4	10.0	Morikawa et al., 2001
	8.4	16.3^**^	Miyoshi et al., 2009
Pineal gland	23.5^a^	18.9^a^	Morikawa et al., 2001
Cerebellum^#^	11.7	168.9^**^	Morikawa et al., 2001
	3.7	134.1^**^	Miyoshi et al., 2009
Medulla oblongata^#^	8.7	102.8^**^	Morikawa et al., 2001
	13.8	125.7^**^	Miyoshi et al., 2009
Spinal cord^#^	6.4	58.1^**^	Miyoshi et al., 2012
Serum	2.1	11.6^**^	Morikawa et al., 2001
	2.5	5.9^**^	Miyoshi et al., 2009
	3.7	10.8^b^^**^	Sasabe et al., 2014b
Plasma	3.5	10.8^c^	Rais et al., 2012
Urine	54.1	486.1^**^	Miyoshi et al., 2009
Liver	21.9	31.7	Miyoshi et al., 2009
Kidney^#^	15.0	179.9^**^	Miyoshi et al., 2009
Pancreas	34.8	93.4^**^	Miyoshi et al., 2009

*The literature since 2001 has been compiled in this table. The values represent means and are obtained from ddY/DAO^−^ mice unless otherwise stated*.

a *Values represent pmol/whole tissues*.

b *Values obtained from serum of ^B6^DAO^−/−^mice*.

c *Values obtained from plasma of DAO-KO mice*.

# *denotes tissues where DAO is present*.

** *P < 0.01 compared with wild-type mice*.

Labrie et al. (2009) also observed that D-serine levels in the prefrontal cortex and amygdala were not different between wild-type C57BL/6 mice and *Dao1*^*G181R*^ mice lacking DAO activity, but that D-serine levels were slightly higher in the hippocampus and significantly higher in the cerebellum of *Dao1*^*G181R*^ mice. Labrie et al. (2010) again observed that the *Dao1*^*G181R*^ mutation caused an increase in D-serine concentration in whole-brain tissues.

In addition to hindbrain, increases in D-serine levels were observed in the urine (Asakura and Konno, 1997; Miyoshi et al., 2009), serum (Nagata et al., 1992; Hashimoto et al., 1993; Morikawa et al., 2001; Miyoshi et al., 2009), kidney (Nagata et al., 1992; Miyoshi et al., 2009), and pancreas (Miyoshi et al., 2009) of ddY/DAO^−^ mice (Table 4).

Rais et al. (2012) also observed that plasma D-serine levels were approximately three times higher in DAO-KO mice than in wild-type mice (Table 4). The D-serine level in the cerebellum was also higher in the DAO-KO mice, but the level in the cortex was not different between the DAO-KO and wild-type mice. Oral administration of D-serine to the wild-type mice and DAO-KO mice showed that D-serine rapidly disappeared from the plasma of the wild-type mice, whereas it remained for a long time in the plasma of the DAO-KO mice (Rais et al., 2012).

Sasabe et al. (2014b) also observed an increase in D-serine content in the serum and urine of ^B6^DAO^−/−^ mice lacking DAO activity compared with wild-type C57BL/6J mice (Table 4).

Many DAO inhibitors have been developed and used to block the degradation of administered D-serine, or to increase the D-serine contents in the brain for therapeutic purposes (Ferraris and Tsukamoto, 2011; Hopkins et al., 2013; Sacchi et al., 2013). Administration of a DAO inhibitor to mice increased D-serine levels in the cerebellum but not in the forebrain of wild-type mice (Strick et al., 2011). Continuous intravenous infusion of a DAO inhibitor elevated the plasma D-serine level for a long time (Rojas et al., 2016). In C57BL/6 mice treated with a DAO inhibitor in their drinking water for three days, D-serine levels were increased in the plasma and kidney, but not in the brain, when compared with control mice (Sershen et al., 2016). Co-administration of D-serine with a DAO inhibitor increased plasma D-serine levels and delayed the disappearance of D-serine from the plasma compared with D-serine administration alone (Alt et al., 2011; Rais et al., 2012; Hin et al., 2015, 2016; Rojas et al., 2016). As well as in plasma, extracellular D-serine levels in the frontal cortex (Hashimoto et al., 2009) and D-serine levels in the spinal cord (Lu et al., 2012) were elevated in mice treated with D-serine plus a DAO inhibitor, compared with mice treated with D-serine alone. The experiments using DAO inhibitors indicate that D-serine is a substrate of DAO, and that it is indeed degraded in mice. However, DAO inhibitors have not always produced the expected effects of increasing D-serine levels in plasma, brain, or spinal cord (Lu et al., 2012; Matsuura et al., 2015; Sershen et al., 2016).

It has been shown that a large amount of D-serine is present in the cerebellum during the neonatal period in rodents. However, DAO gradually accumulates in this brain region with development, and, accordingly, D-serine disappears from the cerebellum as mice mature (Wang and Zhu, 2003; Kim et al., 2005; Kakegawa et al., 2011). These findings also support D-serine being the physiological substrate of DAO. D-Serine is necessary for the development and function of the cerebellum (Kim et al., 2005; Kakegawa et al., 2011).

### D-leucine

Compared with ddY/DAO^+^ mice, ddY/DAO^−^ mice have much higher levels of D-leucine in their serum and brain regions (cerebrum, hippocampus, hypothalamus, pituitary gland, pineal gland, cerebellum, and medulla oblongata) (Table 5; Hamase et al., 2001). This pattern is quite similar to that of D-alanine. However, the absolute amount of D-leucine is approximately 1/10 that of D-alanine. An increase in D-leucine in the blood is considered to be the cause of the increase in the brain. The mouse diets used in the experiments contained a trace amount of D-leucine (Hamase et al., 2001), which may have been the original source.

**Table 5:** Amounts of D-leucine in the brain regions and serum of wild-type mice and mutant mice lacking DAO activity.

**Tissues or physiological fluids**	**Wild-type mice (nmol/g or mL)**	**Mutant mice (nmol/g or mL)**
Cerebrum	0.41	4.15^**^
Hippocampus	0.39	3.52^**^
Hypothalamus	0.55	4.75^**^
Pituitary gland	0.76	5.97^**^
Pineal gland	1.94^a^	4.91^a^^**^
Cerebellum^#^	0.24	5.02^**^
Medulla oblongata^#^	0.27	4.05^**^
Serum	0.39	10.4^**^

*The values represent means and are obtained from ddY/DAO^−^ mice (Hamase et al., 2001)*.

a *Values represent pmol/whole tissues*.

# *denotes tissues where DAO is present*.

** *P < 0.01 compared with wild-type mice*.

### D-proline

Increased levels of D-proline were also observed in the urine, serum, kidney, and pancreas of ddY/DAO^−^ mice compared with ddY/DAO^+^ mice (Nagata et al., 1992; Hamase et al., 2001, 2006b). It is considered that the increase of D-proline in the blood caused the accumulation of D-proline in those organs and excess urinary excretion. However, the absolute amounts of D-proline were very small. At least part of the D-proline observed may have been derived from the diet (Hamase et al., 2001, 2006b).

### D-phenylalanine, D-tyrosine, and D-citrulline

Urine of B6DAO^−/−^ mice lacking DAO activity was found to contain more D-phenylalanine, D-tyrosine, and D-citrulline compared with wild-type C57BL/6 mice (Koga et al., 2016a,b). The amounts of these d-amino acids are trace, and their sources are not still clear.

### D-3,4-dihydroxy phenylalanine

Chiral conversion of D-3,4-dihydroxy phenylalanine (D-dopa) to L-dopa occurred in the kidney homogenates of ddY/DAO^+^ mice, and addition of a DAO inhibitor completely inhibited this conversion (Wu et al., 2006). In contrast to the above homogenates, the kidney homogenates of ddY/DAO^−^ mice could not convert D-dopa to L-dopa. However, the failure of chiral conversion of D-dopa was restored by the addition of DAO preparation, which was again blocked by a DAO inhibitor. Other experiments showed that dopa transaminase is also required for this conversion. This chiral conversion was found to proceed through two steps: D-dopa is converted by DAO to 3,4-dihydroxy phenylpyruvate, which is then reaminated by dopa transaminase to L-dopa (Wu et al., 2006).

### *N*^G^-nitro-D-arginine

After intravenous bolus injection of *N*^G^-nitro-D-arginine (D-NNA), the L-isomer [*N*^G^-nitro-L-arginine (L-NNA)] appeared in the plasma of Swiss mice and ddY/DAO^+^ mice, but not in that of ddY/DAO^−^ mice (Xin et al., 2010). When D-NNA was incubated in the kidney homogenates, L-NNA appeared in the homogenates of the Swiss mice and ddY/DAO^+^ mice, but not in those of the ddY/DAO^−^ mice. However, when a DAO preparation was added to the kidney homogenate of the ddY/DAO^−^ mice, L-NNA appeared in the homogenate. These results indicated that DAO was indispensable in the conversion of D-NNA to L-NNA. Combined with other experiments, they showed that D-NNA was oxidatively deaminated by DAO to *N*^G^-nitroguanidino-2-oxopentanoic acid, followed by stereospecific reamination of the 2-oxoacid by transaminase to produce L-NNA (Xin et al., 2010).

### D-tryptophan

Notarangelo et al. (2016) recently found that injection of D-tryptophan into FVB/N mice led to the appearance of newly formed L-tryptophan in the plasma, liver, forebrain, and cerebellum. This conversion was prevented by a DAO inhibitor. Like other cases above, D-tryptophan is converted by DAO to indole-3-pyuvate, which is then asymmetrically reaminated by aspartate aminotransferase to L-tryptophan. The authors consider intestinal bacteria to be a source of D-tryptophan in this conversion.

### D-cysteine

Shibuya et al. (2013) found in mice that D-cysteine was converted by DAO to 3-mercaptopyruvate, which was further metabolized by 3-mercaptopyruvate sulfurtransferase to produce pyruvate and hydrogen sulfide. The fate of the 2-oxo acid, which was produced by the DAO reaction, was different from its fate in the above three cases. Since hydrogen sulfide has a cytoprotective effect, administration of D-cysteine to mice could alleviate renal ischemia-reperfusion injury (Shibuya et al., 2013).

### D-propargylglycine

When D-propargylglycine was intraperitoneally injected into ddY/DAO^+^ and ddYDAO^−^ mice, polyuria, glycosuria, and aminoaciduria were observed in ddY/DAO^+^ mice, but not in ddY/DAO^−^ mice (Konno et al., 2000). Microscopic observation revealed degenerative and necrotic cells in the straight part of the proximal tubules of the kidneys of the ddY/DAO^+^ mice. This was because D-propargylglycine is a suicide substrate of DAO and irreversibly inactivates this enzyme (Horiike et al., 1975). It is not clear, however, how the inactivation of DAO causes necrosis of proximal tubule cells where DAO is localized. Regardless, ddY/DAO^−^ mice were entirely free from the necrotic action of D-propargylglycine.

### Nutrition

Some d-amino acids are utilized as nutrients in place of the corresponding l-amino acids in animals. Friedman (2010) summarized nutritional potencies of d-amino acids relative to the corresponding l-amino acids in the growth of mice: D-methionine (80%), D-phenylalanine (52%), D-tryptophan (25%), D-histidine (9%), D-valine (5%), D-threonine (3%), and D-isoleucine (1%). These results mostly relate to the substrate specificity of mouse DAO (Konno et al., 1982), in addition to the absorption and transportation of d-amino acids. As described above, those d-amino acids are converted to the corresponding 2-oxo acids by DAO and then asymmetrically reaminated to the l-amino acids, which are used as nutrients. Indeed, using stable isotope tracer techniques, Hasegawa et al. (2004) clearly demonstrated that D-[^2^H_7_]leucine was converted to [^2^H_7_]ketoisocaproic acid and further to L-[^2^H_7_]leucine in ddY/DAO^+^ mice, whereas D-[^2^H_7_]leucine was not converted in ddY/DAO^−^ mice. Consistent with these results, ddY/DAO^−^ mice could not utilize D-phenylalanine as a nutrient in place of L-phenylalanine, in contrast to ddY/DAO^+^ mice (Konno and Yasumura, 1984b). The ddY/DAO^−^ mice could not utilize a D-methionine supplement in the mouse diet, and excreted D-methionine into urine (Konno et al., 1988).

### Main physiological substrates

D-Alanine and D-serine are abundantly present in mice and serves as main physiological substrates of mouse DAO. Other d-amino acids exist at relatively very low levels in the body, but are also potential physiological substrates of DAO if they have definite functions, as well as enzymes or metabolic pathways to generate them inside the body.

It has long been speculated that DAO has true substrates other than d-amino acids (Meister, 1965). Some proposals have been presented so far, but these have not been well supported experimentally. However, it is necessary to keep it in mind that there may be as-yet-undiscovered true physiological substrates for DAO.

## Concluding remarks

Recently, the presence of DAO has been determined in the neutrophils, retina, and small intestine of the mouse, in addition to the kidney and central nervous system. DAO degrades d-amino acids, the products of which are sometimes re-utilized for metabolism. It also functions as a member of the biophylaxis system. Since the discoveries that DAO is involved in the modulation of neurotransmission via the degradation of D-serine, and that DAO is implicated in the etiology of neuropsychiatric diseases such as schizophrenia and amyotrophic lateral sclerosis, a number of studies on DAO have been conducted using mice as experimental models. Many DAO inhibitors have been developed and examined for their efficacy in mice.

Sasabe et al. (2014a) found that human DAO has a much wider distribution in the brain regions than mouse DAO. Recently Rojas et al. (2016) found that co-administration of D-serine with DAO inhibitors enhanced the plasma D-serine level in mice, but not in monkeys or dogs. Plasma D-serine levels were almost same after D-serine administration with or without the infusion of DAO inhibitors in monkeys and dogs, in contrast to mice. These findings indicate that the role of DAO in the metabolism of D-serine is different across species, raising serious problems as to whether the information obtained from studies of mouse DAO can be extended to humans and other animals.

## Author contributions

ReK wrote the draft, YM provided experimental data and made the tables, HS collected the literature and materials, KH organized and managed the team, and RyK provided knowledge and advice. All authors discussed and approved the final manuscript.

### Conflict of interest statement

The authors declare that the research was conducted in the absence of any commercial or financial relationships that could be construed as a potential conflict of interest. The reviewer ER and handling Editor declared their shared affiliation.
